# Symmetrical Neuralgia in Diabetes

**Published:** 1881-02

**Authors:** 


Symmetrical Neuralgia in Diabetes.—Dr. Worms, of Paris, has called attention to the occurrence of symmetrical neuralgias in an advanced period of diabetes. He has recorded two examples—one affecting the sciatic nerve, and one the inferior dental—and believes that the symmetry of the affection is a characteristic of this form, as also is its peculiar severity. It does not yield to the ordinary treatment of neuralgia—quinine, morphia, bromide—and the pain varies in intensity with the amount of glycosuria. —*Lancet.*

